# Pure erythroid leukemia, presenting with pancytopenia. Images in Hematology

**DOI:** 10.1002/ccr3.2357

**Published:** 2019-08-13

**Authors:** Yevgeniy Linnik, Bhavina Batukbhai, Eric Loo

**Affiliations:** ^1^ Department of Pathology and Laboratory Medicine Dartmouth‐Hitchcock Medical Center Lebanon NH USA; ^2^ Department of Hematology Norris Cotton Cancer Center Lebanon NH USA

**Keywords:** bone marrow fibrosis, pancytopenia, pure erythroid leukemia, refractory thrombocytopenia

## Abstract

Pure erythroid leukemia is an aggressive form of acute leukemia, presenting with pancytopenia. It is defined as a neoplasm of erythroid lineage without a significant myeloblastic component, representing >80% of marrow, with 30% or more proerythroblasts. The disease has a rapid clinical course with median survival of only 3 months.

First described by Giovanni Di Guglielmo in the early 1920s, pure erythroid leukemia (PEL) is a neoplasm of erythroid lineage without a significant myeloblastic component, representing >80% of marrow cellularity, with 30% or more proerythroblasts.^1^

A 44‐year‐old woman with no previous history of myelodysplasia presented with progressive fatigue and was found to be pancytopenic with leukopenia(WBC 2.98 × 10^3^/μL) with absolute neutropenia(1.04 × 10^3^/μL), anemia(Hgb of 5.7 g/dL), and thrombocytopenia (5 × 10^3^/μL). Peripheral smear showed circulating erythroid precursors (Figure 1A). The bone marrow was 100% cellular(B, insert), replaced by neoplastic erythroid precursors (>90%) and abnormal megakaryocytes(Figure 1B). Erythroid precursors were predominantly distributed in an intrasinusoidal pattern (Figure 1C). Reticulin showed diffuse myelofibrosis (MF2/3; D). The early erythroblasts were negative for CD34 and positive for E‐Cadherin (Figure 1E,F). CD117 was positive in occasional mast cells distributed throughout. A diagnosis of pure erythroid leukemia was rendered, and a Myeloid Seq^®^ NGS panel revealed *TP53* (p.L194H) mutation.

**Figure 1:**
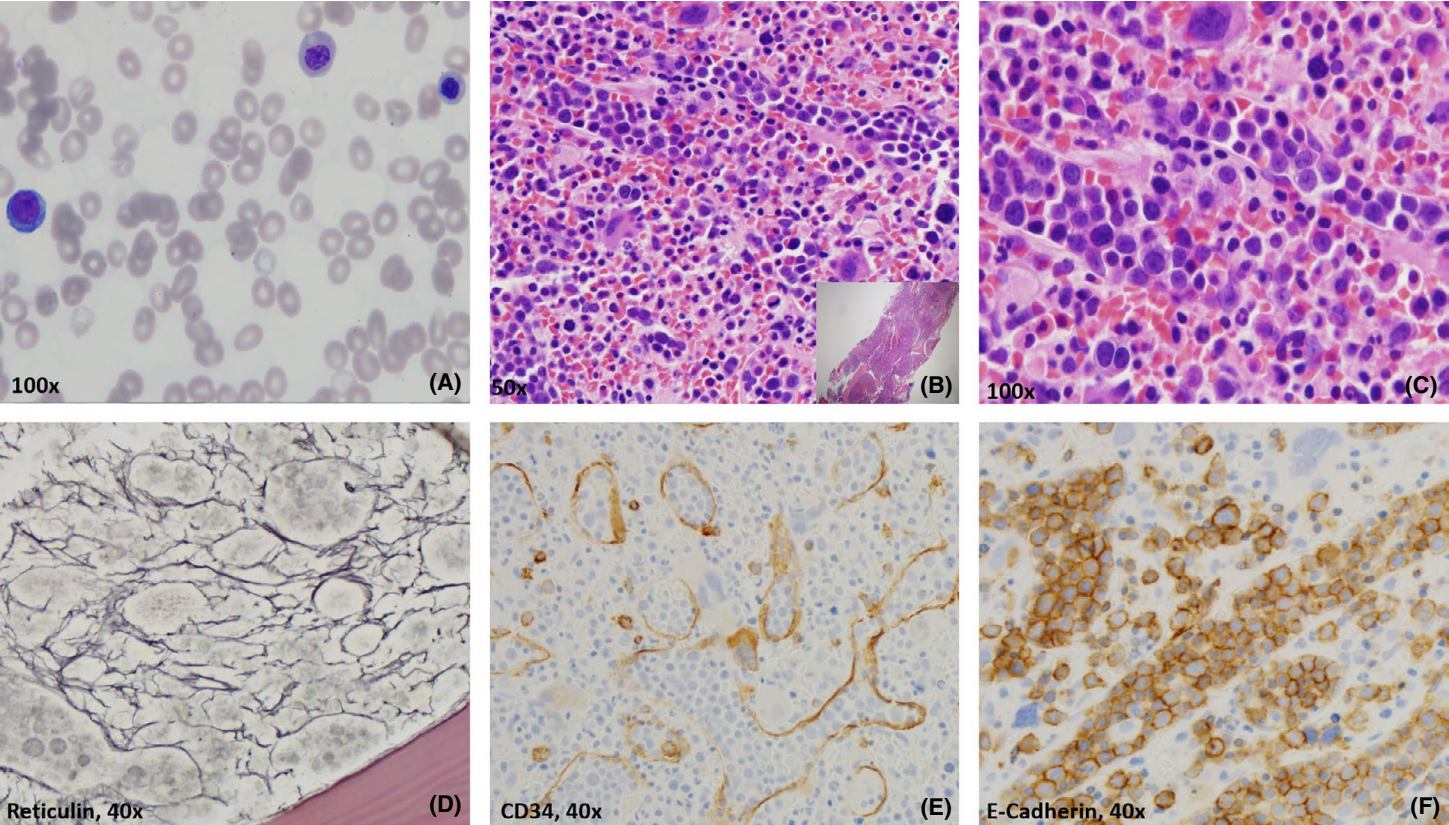
Histopathologic and immunophenotypic findings of pure erythroid leukemia. A, Peripheral blood (PB) smear shows erythroid precursors in various stages of differentiation (Wright‐Giemsa; 1000×). B‐C, Triphene biopsy shows markedly hypercellular marrow, approaching 100% (B, insert 40×) with striking erythroid hyperplasia, representing >90% of the marrow cellularity (B) with medullary sinusoids conspicuously and diffusely dilated, prominently filled with intermediate‐sized pronormoblastic cells with fine chromatin and single nucleoli (C). Reticulin stain highlights dense and diffuse fibrosis (MF two of three; D). The erythroblasts are negative for CD34, which highlights dilated sinusoids (E) and positive for E‐Cadherin (F). B, Hematoxylin and Eosin at 500×; C, Hematoxylin and Eosin at 1000×; D, Reticulin at 400×; E, CD34 at 400×; and F, E‐Cadherin at 400×

The biology of this neoplasm is largely unknown, and the disease has a rapid course with median survival of 3 months.^1^, ^2^

Day 14 biopsy after induction was still involved (>90%) by residual disease. The patient succumbed to the disease after a subdural hemorrhage led to transtentorial herniation.

## CONFLICT OF INTEREST

None declared.

## AUTHOR CONTRIBUTIONS

YL and BB: participated in writing of the manuscript and photomicrographs formatting. EL: made final edits before the submission, as well as taking the microscopic images.
